# Crosstalk Between Retinoic Acid and Sex-Related Genes Controls Germ Cell Fate and Gametogenesis in Medaka

**DOI:** 10.3389/fcell.2020.613497

**Published:** 2021-01-18

**Authors:** Mateus C. Adolfi, Amaury Herpin, Anabel Martinez-Bengochea, Susanne Kneitz, Martina Regensburger, David J. Grunwald, Manfred Schartl

**Affiliations:** ^1^University of Wuerzburg, Developmental Biochemistry, Biocenter, Wuerzburg, Germany; ^2^INRA, UR1037, Fish Physiology and Genomics, Rennes, France; ^3^State Key Laboratory of Developmental Biology of Freshwater Fish, College of Life Sciences, Hunan Normal University, Changsha, China; ^4^Reproductive and Molecular Biology Group, Department of Morphology, Institute of Bioscience of Botucatu, São Paulo State University, Botucatu, Brazil; ^5^Department of Human Genetics, University of Utah, Salt Lake City, UT, United States; ^6^Xiphophorus Genetic Stock Center, Texas State University, San Marcos, TX, United States

**Keywords:** sex determination, retinoic acid, meiosis, gametogenesis, medaka

## Abstract

Sex determination (SD) is a highly diverse and complex mechanism. In vertebrates, one of the first morphological differences between the sexes is the timing of initiation of the first meiosis, where its initiation occurs first in female and later in male. Thus, SD is intimately related to the responsiveness of the germ cells to undergo meiosis in a sex-specific manner. In some vertebrates, it has been reported that the timing for meiosis entry would be under control of retinoic acid (RA), through activation of *Stra8*. In this study, we used a fish model species for sex determination and lacking the *stra8* gene, the Japanese medaka (*Oryzias latipes*), to investigate the connection between RA and the sex determination pathway. Exogenous RA treatments act as a stress factor inhibiting germ cell differentiation probably by activation of *dmrt1a* and *amh*. Disruption of the RA degrading enzyme gene *cyp26a1* induced precocious meiosis and oogenesis in embryos/hatchlings of female and even some males. Transcriptome analyzes of *cyp26a1*–/–adult gonads revealed upregulation of genes related to germ cell differentiation and meiosis, in both ovaries and testes. Our findings show that germ cells respond to RA in a *stra8* independent model species. The responsiveness to RA is conferred by sex-related genes, restricting its action to the sex differentiation period in both sexes.

## Introduction

Sex determination is the decision whether the bipotential gonadal primordium will become a testis or an ovary (Capel, 2000; Devlin and Nagahama, 2002). In vertebrates, this complex and tightly controlled developmental determination process is characterized by a difference in the timing of meiotic initiation (Kimble and Page, 2007; Nishimura and Tanaka, 2014). In multicellular organisms the formation of the gametes is a key event for the production of future generations. In this process the germ cells take two crucial decisions, a temporal one, namely when meiosis entry happens, and a lineage decision to develop either to sperm or egg (Kimble and Page, 2007). However, the timing of the mitosis/meiosis decision and features of meiosis itself are often sex-specific, suggesting a close relationship between the mitosis/meiosis and sperm/egg decisions. In all so far studied vertebrates, initiation of meiosis occurs earlier in females than in males, and retinoic acid (RA) signaling has been assigned a crucial role in this process (Koubova et al., 2006; Bowles and Koopman, 2007; Kimble and Page, 2007).

RA is a vitamin A derivative responsible for activation of several genes during development, being important for the formation of the anterior-posterior axis, and proper development of many different organs and tissues (Sakai et al., 2001). The importance of RA in meiosis entry has been widely studied in mammals and other vertebrates (Smith et al., 2008; Wallacides et al., 2009; Dong et al., 2013). RA is small, polar and diffusible, and the concentration levels are fine-tuned by the balance between its synthesis and its oxidative degradation (Niederreither and Dolle, 2008; Shimozono et al., 2013). The main enzymes involved in the synthesis of RA are the retinaldehyde dehydrogenases (RALDHs). The enzymes responsible for its degradation are the cytochrome P450 family 26 (CYP26s) (Sakai et al., 2001; Yashiro et al., 2004; Deak et al., 2005; Emoto et al., 2005; White et al., 2007). In mice, RA was shown to make primordial germ cells (PGCs) in females entering meiosis by inducing *Stra8* (*stimulated by retinoic acid gene 8*) expression and initiates oogenesis, while testis produce the CYP26B1 enzyme that catalyzes RA degradation, resulting in a delay of meiosis entry in males (Bowles et al., 2006, 2009; Bowles and Koopman, 2010). Therefore, the factors that are regulating the expression of *Cyp26b1* are sex specific. In developing testes, *Cyp26b1* is upregulated by the transcriptional activator SF1 in Leydig cells and by SF1 and SOX9 in Sertoli cells. In ovaries, its expression is suppressed by the female-specific transcription factor FOXL2 (Kashimada et al., 2011).

Teleost is the group of vertebrate with the highest diversity of species and simultaneously sex determination mechanism (Capel, 2017). Complexly, *stra8* is absent in most teleost fish. In species containing *stra8*, the role of RA in meiosis seems to be conserved as in other vertebrates (Feng et al., 2015; Li et al., 2016). In medaka (*Oryzias latipes*), which lacks *stra8*, we have earlier shown that RA is implicated in meiosis regulation in the adult gonad. Expression analyses in embryos of medaka at the SD stage demonstrated that the RA synthesis gene (*aldh1a2*) is expressed in the early somatic gonad of both sexes. However, exogenous treatments with RA did not provide conclusive evidence for RA being involved in inducing the first meiosis as the primary step after SD (Adolfi et al., 2016).

Despite the high diversity of the genetic pathway, the role of RA and the sex-specific timing of germ cells meiosis entry first are conserved in fish and all vertebrates, which motivated to ask the following question: is there any conserved role of RA in the sex determination pathway? To analyze a possible connection between the mechanism of sex determination and meiosis entry we used the well-characterized sex model species medaka. This species has a well-characterized primary male master sex determination gene on the Y-chromosome. This gene, *dmrt1bY*, is a duplication of the autosomal copy, *dmrt1a*, a highly conserved gene generally involved in testis development and differentiation (Matsuda et al., 2002; Nanda et al., 2002). Here, we show that exogenous treatments with RA in early medaka embryos acts as stress factor leading to an increase in expression of important male sex-related genes, therefore blocking meiosis. Disruption of the *cyp26a1* gene induced early meiosis in females and produced some males with transient oocytes in the gonad prior to testis differentiation. Our results indicate for the first time that the sex regulatory network may control the germ cell responsiveness to RA, which in turn regulates the different timing of meiosis entry of female and male in a *stra8* independent model.

## Materials and Methods

### Animals

Laboratory reared medaka (*Oryzias latipes*, Class Actinopterygii, order Beloniformes, family Adrianichthyidae) were used. For detailed description of this model species and its features see (Kinoshita et al., 2009). All experiments were performed with fish of the Carbio strain. The animals were kept under standard photoperiod cycle of 14/10 h light/dark at 26°C (±1°C). Eggs were collected 1–2 h after starting the light cycle and raised at 26°C in Danieau's medium (17.4 mM NaCl, 0.21 mM KCl, 0.12 mM MgSO_4_, 0.18 mM Ca(NO_3_)2, 1.5 mM Hepes, pH 7.2). The stages of development were identified according to Iwamatsu (2004).

Animals for colony breeding and embryo production were kept and sampled in accordance with the applicable EU and national German legislation governing animal experimentation, in particular all experimental protocols were approved through an authorization (568/300-1870/13) of the Veterinary Office of the District Government of Lower Franconia, Germany, in accordance with the German Animal Protection Law (TierSchG).

### *In vivo* Drugs Treatments

Treatments of embryos and dilutions of the drugs were made in Danieau's medium. To investigate an effect on regulation of sex-related genes, we performed long-term treatments from stage 29, before the sex determination period, and kept in the dark until 1 day after hatching (dah), first meiosis entry period in females. AM580 (10 nM), an agonist of the retinoic acid receptor alpha, and *all-trans*-retinoic acid ATRA (10 nM) were added to the medium and medium changed every 2 days. The exclude any effect of stress during the treatments, we co-treated the embryos with or without Metyrapone (5 μM, Sigma-Aldrich), a compound that inhibits endogenous cortisol synthesis. The selected drugs concentration for the treatments were based on previous studies (Adolfi et al., 2016, 2019). Specimen were collected at 1 dah and genotyped for sex by PCR for the Y-linked male determining gene *dmrt1bY* using genomic DNA as template.

### Disruption of Cyp26a1 by TALEN

The genomic sequence of *cyp26a1* (Ensembl gene number ENSORLG00000014516) was retrieved from the Ensembl medaka genome browser (http://www.ensembl.org/Oryzias_latipes). The construction of TALEN expression vectors (left, pCS2TAL3DDD, and right, pCS2TAL3RRR, with both vectors containing the respective TALE fragment, the *Fok*I cleavage domain, and other necessary components) were developed following the standard procedure (Dahlem et al., 2012). The TALEN target sites of *cyp26a1* were designed in the second exon, with the right binding site located at the junction of exon 2 and intron 2. The *cyp26a1* TALEN recognition sequences were left TALEN 5′ –TCTCCAACATGCACGGAT- 3′ and right TALEN 5′ –TGGAGACTCACCTTTTT- 3′. Between the binding sites, an 18 bp spacer is included, where the *Fok*I nuclease cuts.

*In vitro* transcription of TALENs was carried out with the Sp6mMESSAGEmMACHINE Kit (Ambion). The resulting mRNA was purified by phenol/chloroform-extraction and then quantified using NanoDrop-2000 (Thermo Scientific). The left and right arm mRNA of each TALEN pair was then mixed at a molar ratio of 1:1, with a final concentration of 100 ng/μL mRNA of each arm, and stored at −80°C until use. About 200 to 600 pg of the mRNA mixture was directly microinjected into medaka embryos at the one-cell stage. The injected embryos were cultivated at 26°C and 10 animals collected at stage 1 dah to extract DNA for mutation efficiency analysis.

### Genotyping of Embryos and Adult Fish

To determine the genotypic sex of embryos and adult fish and the presence and absence of mutations, genomic DNA was extracted. Caudal fin clips of the adult fish or whole hatchling were incubated for 1 h at 95°C in 100 μL of Base Solution (25 mM NaOH, 0,2 mM EDTA, pH = 12) and shaking. The solution was cooled down on ice, 100 μL of Neutralization Solution (40 mM Tris-HCl pH = 5.0) added and vortexed. Two microliter of the total volume was used in a 25 μL PCR reaction. The PCR products were resolved on 1% agarose gels.

For determination of genotypic sex, a pair of primers (Supplementary Table 1) was used that amplifies fragments of both *dmrt1a* (1,100 bp) and *dmrt1bY* (900 bp), yielding one PCR product (*dmrt1a*) in XX genotypes, and two PCR products (*dmrt1a* and *dmrt1bY*) in XY genotypes. To detect *cyp26a1* TALEN mutants, primers were designed flanking the region where the mutations are expected (exon2). PCR product were purified using GenElute^™^ Gel Extraction Kit (Sigma-Aldrich) according to the manufacturer's instructions and sequenced using the PCR amplification primers.

### Luciferase Reporter Assays

HEK 293 cells were cultured in Dulbecco's modified Eagle's medium and 10% fetal calf serum, and maintained at 37°C, 5% CO_2_ with 100% humidity. To analyze transcriptional regulation of *dmrt1a*, an 11875 bp fragment upstream of the start codon was isolated and cloned into pGL4.20 vector containing the *firefly* luciferase gene (Dmrt1aprom::LucFF) as described (Adolfi et al., 2019). In addition, the responsiveness of the HEK 293 cells to ATRA and AM580 was tested using the pGL3-RARE-luciferase plasmid (Addgene, Cat. 13458), which contains the retinoic acid responsive element (RARE) upstream of the *firefly* luciferase gene.

Transient transfections in HEK 293 were done at 80% confluency by a polyethylenimine-based procedure. The empty pGL4.20 vector containing the tk promoter and the *firefly* luciferase gene (pGL4.20-tkmini) was used as negative control. To normalize *firefly* activity, cells were co-transfected with a *Renilla* luciferase expressing plasmid (pGL4.74) (Regneri et al., 2015).

For luciferase assays, single wells of a 24-well plate were co-transfected with *firefly* and *Renilla* luciferase reporter constructs in a 5:1 molar ratio. The concentration of each construct was calculated in order to obtain a total DNA concentration of 0.5 μg per well. pGL4.20-tkmini and Dmrt1a-prom::LucFF reporter constructs were used with and without co-transfection of the transcriptional activator SF1 of medaka (100 ng). The SF1 expression vector (pcDNA3.1::medakaSF1) was kindly donated from Yann Guiguen (INRA, France). After 16–18 h (day 1), medium was changed. On day 2, cells were incubated for 24 h and with 1 μM ATRA, 10 nM AM580 or DMSO for control. On day 3, cells were harvested in 100 μl of 1 X PLB (Promega).

Renilla and firefly luciferase activities were quantified using the Dual-Luciferase® Reporter Assay System from Promega and the TriStar LB941 microplate multimode reader (Berthold Technologies). Experiments result from at least three replicates and error bars represent the standard error of the mean.

### RNA Sequencing

Three individual ovaries and three pools of three testes from wildtype Carbio strain and *cyp26a1*–/–of medaka were homogenized in TRIzol® reagent (Invitrogen). The total RNA phase was isolated using chloroform and purified using RNeasy® Mini kit (Qiagen) following the manufacturer's instructions. The RNA quality was assessed by measuring the RNA Integrity Number (RIN) using an Agilent 2100 Electrophoresis Bioanalyzer Instrument (Agilent Technologies 2100 Expert). RNA samples with RIN > 8 were used for sequencing.

RNA sequencing libraries were constructed following the standard TruSeq Illumina mRNA library preparation protocol (www.illumina.com; Illumina Inc., BGI, Hong Kong). Read length = 150, sequencing depth for paired end: 65–71 million reads.

### Transcriptome Analysis

Transcriptome sequences were mapped to the *O. latipes* reference genome (Ensembl Release 93) using the RNA-sequence aligner STAR (https://github.com/alexdobin/STAR/releases). Transcripts were quantified as expected read counts using RSEM (http://deweylab.github.io/RSEM). Differentially expressed genes between testis and ovary were detected by DESeq2 (Love et al., 2014) (Bioconductor/R) for wildtype and mutants. Genes were considered to be differentially expressed, if *p*.value <= 0.05 AND log2FC ≤ −2 (higher expression in female) and log2FC ≥ +2 (higher expression in male). Histograms for genes with log2FC > 2 AND baseMean > 100 were plotted and genes showing comparable regulation between male and female wildtype and mutant samples were selected. Functional clustering was made using DAVID Bioinformatics Resources 6.8 (https://david.ncifcrf.gov/).

### Real Time Quantitative RT-PCR

Wildtype and mutant organs of adult (4 months after hatching) males and females and whole embryos of different developmental stages were collected. Total RNA was extracted from 3 pools of adult fish organs (*n* = 4) or whole embryos (*n* = 20) using the TRIZOL reagent (Invitrogen) according to the supplier's recommendation. After DNase treatment, reverse transcription was done from 2 μg total RNA using RevertAid First Strand Synthesis kit (Fermentas) and random primers. Real-time quantitative PCR was carried out with SYBR Green reagent and amplifications were detected with a Mastercycler® ep realplex (Eppendorf). All results are averages of at least three independent RT reactions from three independent RNA preparations. Transcript levels of the target genes were normalized against the medaka elongation factor-1 alpha (*ef1a*) gene (Supplementary Table 1). The ΔCt values presented as means ± standard error of the mean (SEM), were analyzed by one-way ANOVA, Tukey's and Student's *t*-test. A significance level of *P* < 0.05 was used for all tests.

### Light Microscopy

Whole larvae and gonads from adult fish were dissected and fixed in Karnovski solution (2% glutaraldehyde and 4% paraformaldehyde in Sörensen buffer [0.1 M, pH 7.2]) for 24 h at 4°C. Then, samples were washed in water, dehydrated in increasing concentrations of ethanol, and embedded in Historesin Technovit 7100 (Kulzer, Hanau, Germany). Serial sections of 2 μm thickness were obtained and counterstained with hematoxylin & eosin (HE).

## Results

### Induction of Sex Determination Genes After RA Induction

**Figure 1:**
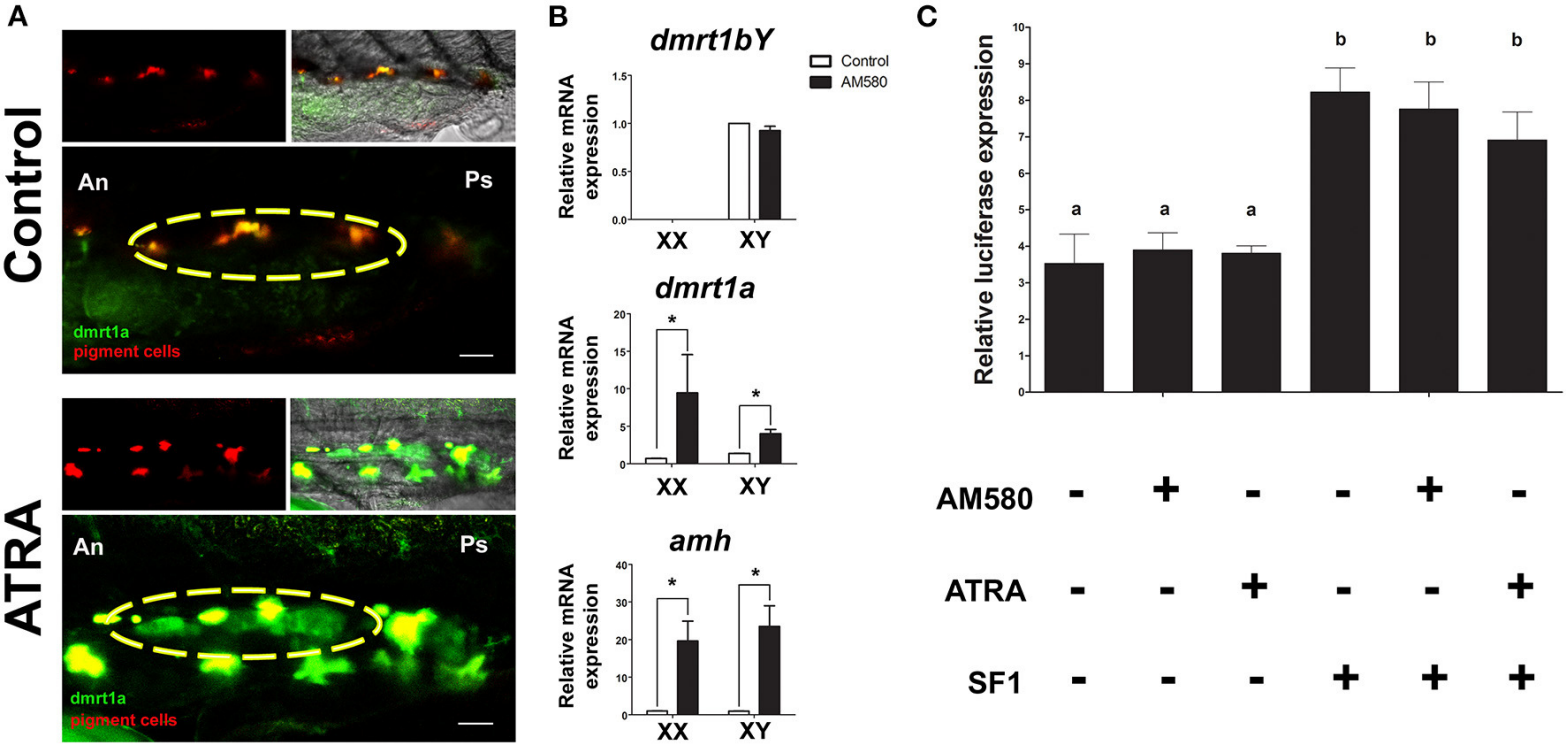
Regulation of sexual development genes after RA pathway activation. **(A)** Long-term treatments of BACdmrt1a::GFP medaka embryos led to gonad (yellow circle) specific induction of *dmrt1a* at 1 dah. **(B)** Expression levels of *dmrt1bY, dmrt1a*, and *amh* at 1 dah after long-term treatments with AM580. No effect on *dmrt1bY* expression is observed, however, significant upregulation of *dmrt1a* and *amh* occurred in embryos of both sexes. Values are expressed as arbitrary mRNA units normalized against the expression levels of *ef1a* amplified from the same template and relative to the average expression of control male and female embryos. Asterisk indicates a significant difference (*p* < 0.05) after Student's *t*-test comparing the expression between control and AM580 treatments. **(C)** The *dmrt1a* promoter activity was higher when co-transfected with medaka SF1, but no significant luciferase signal was observed after treatments with both ATRA and AM580. Scale bar = 60 μm.

We performed treatments of medaka embryos at different time points with ATRA and AM580 to activate the RA pathway. From the treated embryos, we analyzed expression of genes known to be involved in sex determination or gonad differentiation. Long-term treatments (stage 29 until 1 dah) of BACdmrt1a::GFP transgenic fish with ATRA resulted in a strong induction of reporter gene expression exclusively in the somatic gonad at hatching stage in both sexes (Figure 1A). Gene expression levels of male-related genes were determined from whole embryos after long-term treatment with AM580 (Figure 1B). The *dmrt1bY* expression levels were unaffected in males. However, *amh* and *dmrt1a* showed significantly increased mRNA levels in both sexes.

To date, the responsiveness of *dmrt1a* to RA is unknown. Hence, to check whether the treatments had a direct effect by activating *dmrt1a* transcription, we analyzed the 11,8 kb promoter of *dmrt1a* after treatments with ATRA or AM580 in HEK 293 cells. The HEK 293 cells were shown to be capable to respond to both ATRA and AM580 when compared to control (DMSO), indicating that the retinoic acid receptors (RAR/RXR) are endogenously produced in this cell line (Supplementary Figure 1). Cells co-transfected with the cofactor SF1 showed a significant increase of the *dmrt1a* promoter activity. However, treatments with RA had no effect on the promoter of *dmrt1a in vitro* (Figure 1C).

### Cyp26a1–/–Medaka Gonad Development

We showed earlier that *cyp26a1* is differently expressed in gonads of medaka, with much higher transcript levels in females than in males (Adolfi et al., 2016) This indicated that *cyp26a1* in medaka may perform the role of *CYP26B1* in mammals. To evaluate a possible role of *cyp26a1* in the timing of meiosis entry and consequently in sex determination/differentiation, we generated knockout *cyp26a1* medaka.

To molecularly characterize the *cyp26a1* TALEN induced mutations, we amplified the expected target site from F_0_ embryos and adult male and female founders and sequenced the 544 bp PCR product (Supplementary Figure 2A). We obtained three different mutant lines (Supplementary Figure 2B) with deletions at the target site. However, only the Δ5 mutation conceptually translates into a protein with a predicted compromised function (frameshift and, premature termination), while the other two presented an in-frame deletion of two or three aa that still could lead to fully functional enzyme (Supplementary Figure 2C). All three mutants present deletions or substitutions within the P450 superfamily domain, however, the Δ5 mutation is predicted to translate into a shorter protein that lacks the Cytochrome P450 cysteine heme-iron ligand signature. Therefore, we generated *cyp26a1*–/– animals and performed the mutants analyzes only from the Δ5 mutation line.

**Figure 2:**
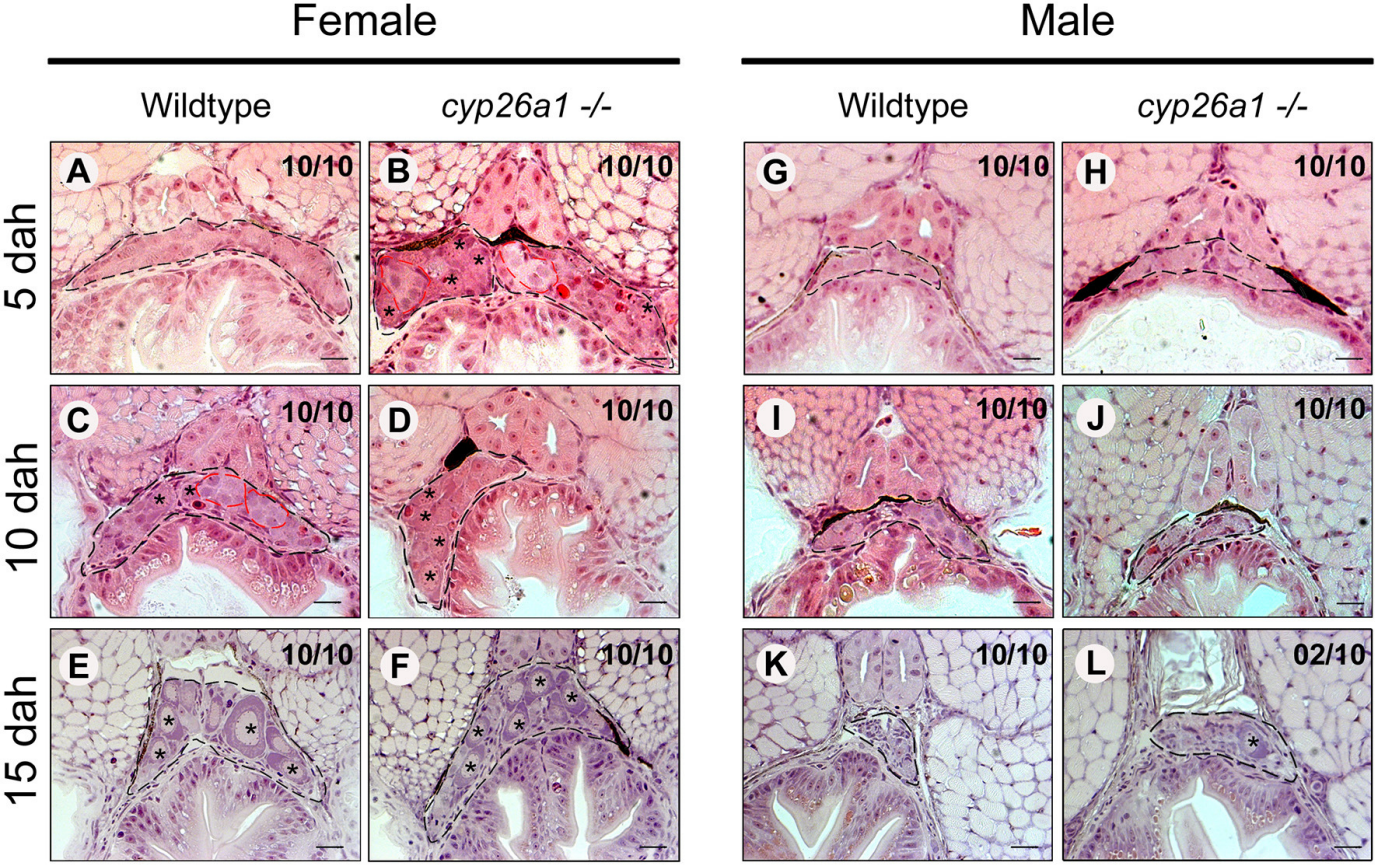
Germ cell proliferation and differentiation during male and female gonad development in wildtype and *cyp26a1*–/–medaka. Gonad (black dashed lines) of *cyp26a1*–/–female present higher amounts of differentiated germ cells (red dashed lines) at 5 days after hatching (dah) in comparison with the wildtype **(A,B)**. At 10 dah, differentiated germ cells are more preeminent in the wildtype female, while in the mutant, the higher amount of pre-vitellogenic oocytes (star) indicates more advanced stage of oogenesis **(C,D)**. At 15 dah, no apparent differences were observed in female gonads **(E,F)**. In males, no differences were observed between wildtype and mutant at 5 dah **(G,H)** and 10 dah **(I,J)**. At 15 dah, no sign of germ cells differentiation is observed, by comparing with the wildtype gonad **(K,L)**. However, some *cyp26*–/–males presented pre-vitellogenic oocytes (star). Scale bar = 20 μm.

We then followed the gonad development of wildtype and *cyp26a1*–/–in male and female larvae at the early meiosis stages (Figure 2). Already at 5 dah, differences in the germ cells are observed in females, in which the *cyp26a1*–/– present more proliferating germ cells compared to the wildtype (Figures 2A,B), while in male no morphological differences were observed until 15 dah (Figures 2G–J). Mutant females at 10 dah apparently contain more pre-vitellogenic oocytes than wildtype females, indicating increased oogenesis and meiosis entry in the mutant at this stage (Figures 2C,D). At 15 dah, the gonads of both wildtype and *cyp26a1*–/–females presented no apparent morphological difference anymore (Figures 2E,F). Strikingly, 2 out of 10 15 dah males of *cyp26a1*–/–had an isolated pre-vitellogenic oocytes inside the undifferentiated gonad, and no sign of germ cell proliferation could be observed (Figures 2K,L). Comparing 4 months old wildtype and mutant mature gonads of medaka no apparent differences in morphology were observed in both sexes (Supplementary Figure 3). Despite the development of oocytes at 15 dah in males of *cyp26a1*–/– genotype, no sign of any female structure was observed in adult testis.

### Treatments of Wildtype and Cyp26a1–/–Medaka Embryos

**Figure 3:**
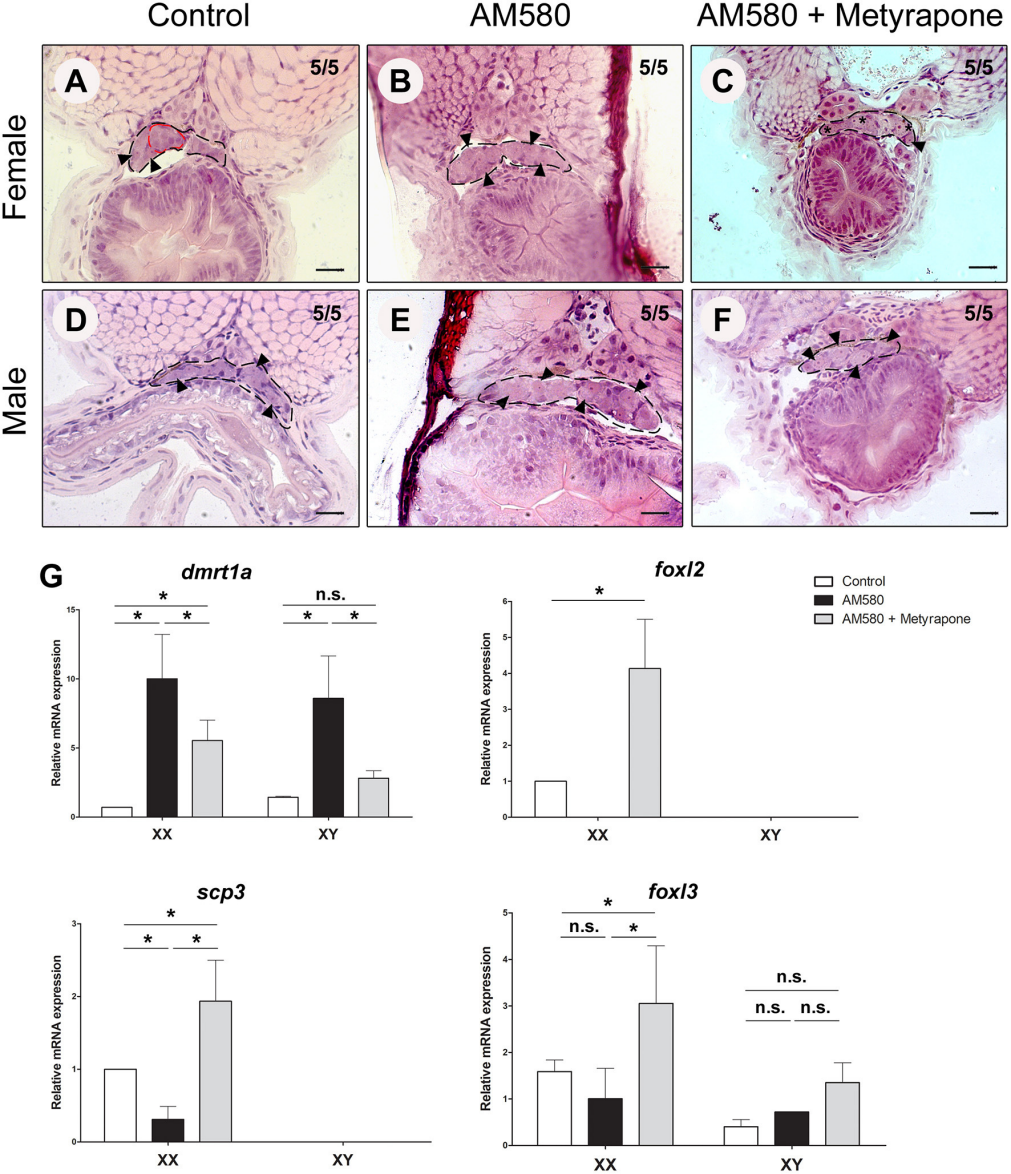
Exogenous treatment of AM580 and Metyrapone in *cyp26a1*–/–embryos during the sex determination period. Gonad (black dashed lines) of *cyp26a1*–/–female containing differentiated germ cells (red dashed lines) at 1 days after hatching (dah) in the control embryos **(A)**. After treatments, XX embryos present only undifferentiated germ cells (arrowhead) **(B)**. Co-treatment with AM580 and Metyrapone showed increase of early oogenesis stage germ cells (star) in females **(C)**. No morphological differences in the gonads of XY mutants were observed in the control **(D)** and in treated embryos with AM580 **(E)** and both AM580 and Metyrapone **(F)**, all containing only undifferentiated germ cells (arrowhead) Scale bar = 20 μm. Expression of *dmrt1a, foxl2, foxl3*, and *scp3* in *cyp26a1*–/–embryos after treatment with AM580 and Metyrapone. After Student's *t*-test (*p* < 0.05), significant (asterisk) and not significant (n.s.) expression differences were observed between control and treatment groups **(G)**.

We performed treatments of *cyp26a1* KO embryos with AM580 from embryo stage 29 until 1 dah. Control females of *cyp26a1*–/–medaka presented morphologically differentiated germ cells already at 1 dah, showing commitment to gametogenesis (Figure 3A). However, XX mutants treated with AM580 had only undifferentiated germ cells (Figure 3B). The developing gonads of both control and treated XY mutants show no sign of germ cell differentiation (Figures 3D,E), indicating that AM580 delays germ cell commitment to gametogenesis in *cyp26a1* mutants.

Retinoic acid is an important morphogen, and treatments with AM580 performed during the sex determination period led to several malformations in the embryos (data not shown). Our recent study showed that temperature stress and cortisol treatments lead to masculinization of XX medaka, possibly through a direct activation of *dmrt1a* in the gonad and repressing germ cell differentiation (Adolfi et al., 2019). Hence, such stress factors in our treatments could possibly interfere with the effect of AM580 in meiosis initiation. To test this hypothesis, we co-treated the *cyp26a1* KO embryos with AM580 and Metyrapone, a compound that inhibits the endogenous production of cortisol. Early differentiating oocytes (stage I) were observed in treated females (Figure 3C), and only undifferentiated germ cells were present in males in the same conditions (Figure 3F) showing that RA leads to germ cell differentiation in females.

Expression analyzes of mutant embryos showed that treatments with AM580 resulted in presented upregulation of the male-related gene *dmrt1a* in both sexes, while the female-related *foxl2* gene expression is extremely reduced in females (Figure 3G). Treatments with both AM580 and Metyrapone presented less *dmrt1a* expression compared to those treated with AM580 alone, while *foxl2, foxl3* (oogenesis inducer) and the meiosis marker *scp3* were upregulated in females when compared to the control (Figure 3G).

### Transcriptome Analyzes of Adult Cyp26a1–/–Medaka Gonads

**Figure 4:**
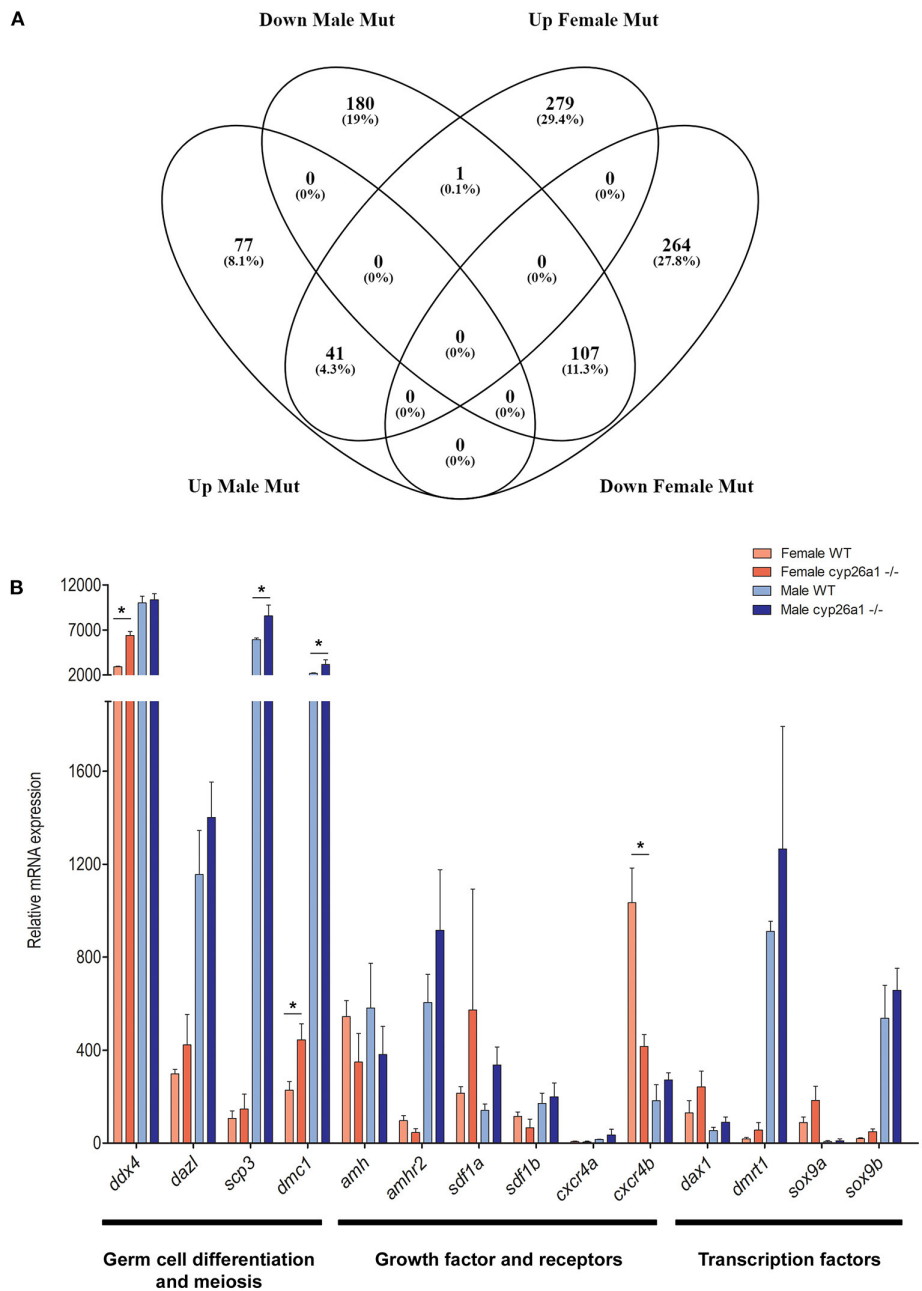
RNA-seq analyzes of adult gonad of wildtype and *cyp26a1*–/–medaka. **(A)** Venn diagram showing the number of regulated genes in both male and female mutants compared to the wildtype gonads. **(B)** Expression levels of sex-related genes known to be involved in germ cell migration, proliferation, differentiation and meiosis. Asterisk indicates a significant difference (*p* < 0.05) after Student's *t*-test.

Despite the effect in the early gonad of the *cyp26a1* mutants, no apparent effect was seen in the adult animals on the cellular level. Nevertheless, the transcriptome analyses showed significant differences between adult wildtype and KO gonads of both male and female (Figure 4). Differential gene expression analysis (LogFC > 2) revealed that the mutation of *cyp26a1* regulated more genes in female than male gonads (Figure 4A; Supplementary Table 2).

The mutant ovaries presented higher number of exclusive upregulated genes (279), some of which are related to retinoic acid metabolism (e.g., *aldh1a2, aldh8a1*, and *rarb*), and gametogenesis (*spata7, aqp3a*, and *zar1*). In the genes exclusively downregulated in mutant females (264), some factors related to the TGF-beta signaling (*smad1, inhbb*, and *lefty1*), retinol metabolism and steroidogenesis (*cyp1a*) were affected. Interestingly, the *gata4* gene, known to be required for gonad formation and testis development in mice (Hu et al., 2013), was strongly downregulated. Despite of a fewer number of genes regulated exclusively in male mutants, the *nr4a1* gene, important in testis function (Daems et al., 2014), was highly upregulated.

The genes that were downregulated in the mutant gonads of both sexes (107) are enriched for functions related to mitochondrial electron transport (e.g., *cox1, cox2*, and *cox7a2*) and response to oxidative stress (e.g., *rsp29*). On the other hand, the genes that were upregulated in the mutants of both sexes (41) are related to immune response (e.g., *c6*) and peptidase activity (e.g., *cela1, ela2*, and *prss1*).

The induction of sex-related genes by AM580 treatments already indicated a possible effect of RA on gametogenesis and germ cell differentiation. This could also be observed by the genome wide expression analysis: genes which have a crucial function in germ cell differentiation (*ddx4* and *dazl*) and meiosis (*scp3* and *dmc1*) were slightly upregulated in the mutants of both sexes (Figure 4B). Similarly, growth factors (e.g., *amh*/*amhr2*, and *sdf1/cxcr4*) and transcription factors (e.g., *dmrt1* and *sox9*) related to germ cell differentiation, proliferation and survival were regulated in mutants, especially in females (Figure 4B).

## Discussion

The network and factors involved in sex determination appear to be more complex and diverse than previously thought (Herpin and Schartl, 2015). Recent studies suggest that neither the master sex determination gene nor the downstream regulatory network of gonad determination is conserved (Herpin et al., 2013). However, the timing of meiosis entry is conserved in vertebrates, with PGCs proliferating in female embryos first and then enter meiosis immediately afterwards, much earlier than in males (Bowles and Koopman, 2007; Morinaga et al., 2007; Saito and Tanaka, 2009; Wallacides et al., 2009). Therefore, to characterize the relation between the action of the sex determining genetic network and meiosis entry and a possible role of retinoic acid in this process, we performed treatments with RA agonists and analyzed possible changes in the process and sex determination.

At the sex determining stage, the male-related genes *dmrt1a* and *amh* were induced by RA treatment in both sexes. Overexpression of *amh* in both sexes after AM580 treatment can also be correlated to blocking of germ cell differentiation, since AMH signaling acts in the supporting cells of the undifferentiated gonad of medaka to promote proliferation of the mitotically active germ cells, which subsequently enter meiosis (Nakamura et al., 2012). Importantly, the upregulation of *dmrt1a* occurs specifically in the gonad, and this gene is known to have a main role in testis differentiation and maintenance (Masuyama et al., 2012). We showed recently that early induction of *dmrt1a* in medaka embryos results in sex-reversed XX males and blocks germ cell proliferation and differentiation (Adolfi et al., 2019). In medaka, the mechanism by which *dmrt1a* and *dmrt1bY* block germ cell differentiation is unknown, but in mice it has been shown that DMRT1 restricts RA responsiveness by repressing *Stra8* transcription, preventing meiosis and promoting spermatogonial development in adult testis (Matson et al., 2010, 2011; Krentz et al., 2013). However, medaka has no *stra8* gene, suggesting that *dmrt1* would still have the same role for the germ cells, but through a different mechanism than in mammals. It was hypothesized that DMRT1 and retinoic acid receptor (RARα) are antagonistic regulators of some key feminizing genes (Huang et al., 2017), indicating that the presence of DMRT1 in the germ cells would be regulating their responsiveness to RA.

We have previously demonstrated that activation of the RA pathway could not induce meiosis in early embryos, but only in gonads that already started sexual differentiation and gametogenesis (Adolfi et al., 2016). Here we demonstrate that the same activation leads to overexpression of genes related to inhibition of germ cell differentiation (*amh* and *dmrt1a*) in the gonad, preventing the germ cells to differentiate and enter meiosis. The *dmrt1a* promoter activity experiments showed that treatments with ATRA and AM580 had no significant effect on the promoter activity of *dmrt1a*, indicating that RA upregulates *dmrt1a* only indirectly but not by direct transcriptional activation *in vivo*.

We showed that exogenous RA treatments can increase both *cyp26a1* and *cyp26b1* expression, the former being more sensitive to the treatments (Adolfi et al., 2016). This could lead to degradation of the drug and camouflage its direct effect. In order to remove the possible effect of *cyp26a1* upregulation in the gonad, and increase the effect of RA in germ cell differentiation, we generated a full KO of the *cyp26a1* gene. A *cyp26a1* deficiency in Tilapia and catfish did not affect normal development of the animals and induced earlier initiation of meiosis for both XX and XY fish, but it was still earlier in females than in males (Feng et al., 2015; Li et al., 2016). Similarly, we observed more noticeable germ cell in meiosis, and early oogenesis in female *cyp26a1* knockouts, but only after the sex determination stage. Despite the minor effect noted in adult gonads, the transcriptome analyzes show that ovaries of *cyp26a1*–/– medakas presented more regulated genes than other groups, since wildtype ovaries had the highest expression of *cyp26a1* compared to other tissues (Adolfi et al., 2016; Biscotti et al., 2018). Consistently, strong upregulation was observed in mutant ovaries of genes known to be related to gametogenesis regulation like *zar1, spata7*, and *aqp3a*, the latter two genes already being known to be regulated by retinoic acid (Liu et al., 2005; Huang et al., 2006; Bellemere et al., 2008; Wang et al., 2009). The *zar1* gene was described to have a conserved evolutionary role in ovarian follicle development and in the oocyte-to-embryo transition, but not correlation between RA and *zar1* was demonstrated so far (Wu et al., 2003).

Interestingly, strong downregulation of *gata4, ihnbb* and *cxcr4b* in mutant ovaries was observed. The *gata4* gene was reported to be a key transcriptional regulator of ovarian somatic cell function in both fetal and adult mice (Kyronlahti et al., 2011; Efimenko et al., 2013). In addition, *gata4* was proposed to be important for gonadal development and maturation in both sexes of Tilapia (Li et al., 2012). Analyzes *in vitro* demonstrated that *Gata4* is upregulated in murine embryonic stem cells (ESCs) after ATRA treatments (Mauney et al., 2010). The *inhbb* gene is known to play a role in regulating steroid hormone production during follicular development (Luisi et al., 2005), and testis of vitamin A-deficient rats showed low levels of the Inhibin alpha-subunit, which increases after retinol administration (Zhuang et al., 1997). The SDF1/CXCR4 signaling is known to be required for the maintenance of mouse spermatogonial stem cells, and inhibition of CXCR4 signaling increases the responsiveness of the germ cells to RA (Yang et al., 2013). In addition, study in Orange-spotted grouper demonstrated that the expression of *cxcr4b* gene is sharply decreased in mature ovary (Lu et al., 2018). In *cyp26a1* mutant testis, the orphan nuclear receptor 4A1 (*nr4a1*) was strongly upregulated. This transcription factor can heterodimerize with the retinoid X receptor (RXR) (Zetterstrom et al., 1996). In mammals, NR4A1 is strongly and rapidly induced in Leydig cells, and it regulates several steroidogenic genes including *Star, Hsd3b1*, and *Cyp17a1* (Daems et al., 2014).

The early meiosis entry in *cyp26a1*–/–females, together with the regulation of genes related to gametogenesis in adult mutants, confirm the role of RA in germ cell differentiation after the sex-determining period. However, the timing of meiosis entry is still different between male and female, being much later in males. Interestingly, germ cells of some males entered oogenesis around the period of male meiosis initiation. However, neither sex reversals nor ovotestes were observed in the adult gonad. This striking result demonstrates a tight correlation between RA in regulating gametogenesis and possibly germ cells sex identity in a *stra8* independent model species. The formation of the testis and absence of oocytes in adult *cyp26a1*–/–males could be related to the late initiation of the male sex differentiation pathway, which occurs around 30–45 dah (Nishimura and Tanaka, 2014), and overrides the initiation of female gametogenesis. Testicular germ cell transplantation into female undifferentiated embryonic gonad of rainbow trout produces functional egg, demonstrating the outstanding capacity of the germ cell to respond to the gonad environment (Okutsu et al., 2006). Hence, we hypothesize that, in for those few *cyp26a1 –/–* medaka, the germ cells start oogenesis until the time of testicular differentiation period, which leads to the development of normal male gonads and regression of the already formed oocytes.

**Figure 5:**
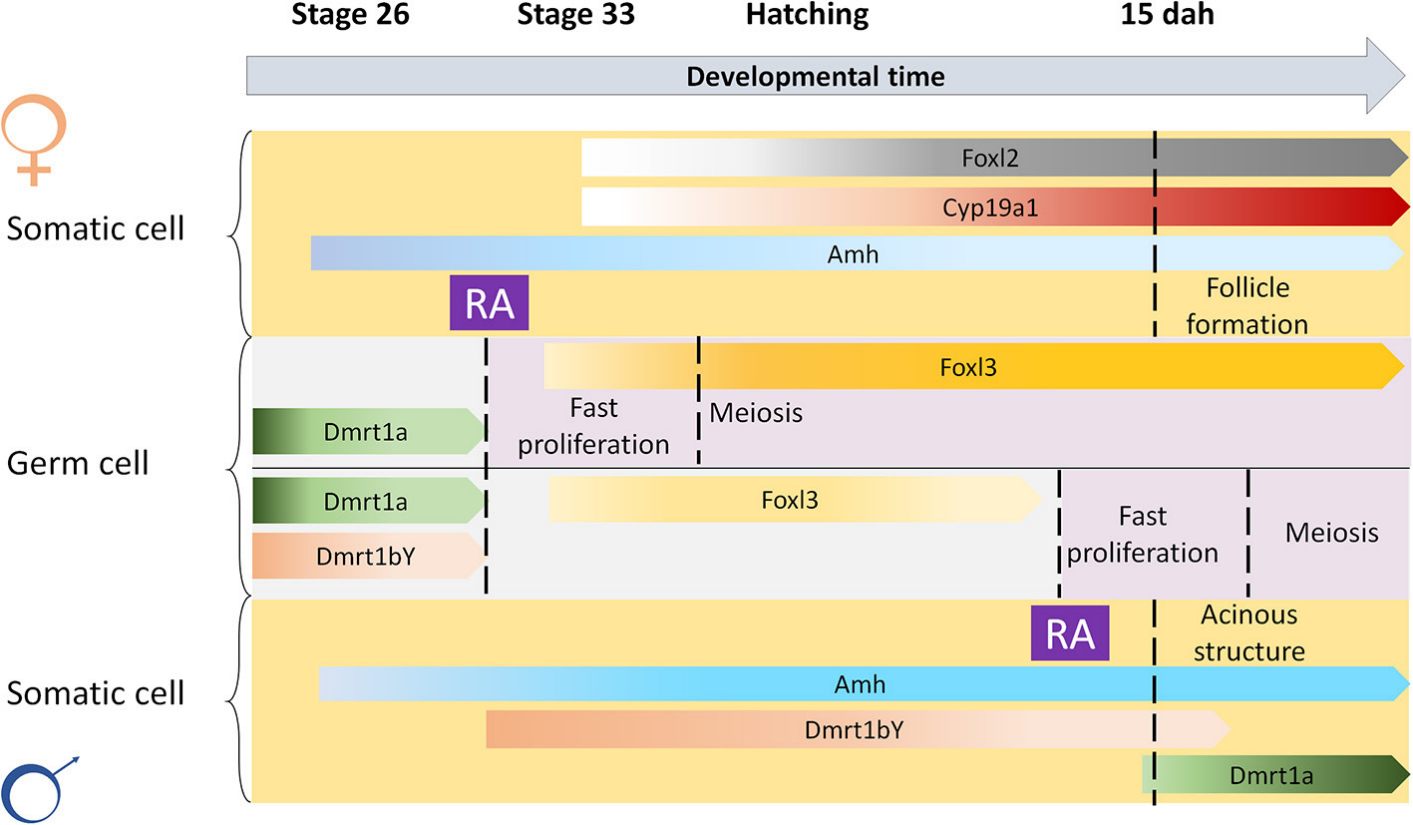
Scheme for spatial-temporal crosstalk between retinoic acid and sex-related genes in meiosis induction. Retinoic acid (RA) is important to induce gametogenesis in both sexes. The restrain of RA action at early stages in the germ cells of males is possibly regulated by male-related factors such as Dmrt1a, Dmrt1bY, or Amh, that are known to inhibit germ cell differentiation.

Treatments with AM580 were expected to strongly induce gametogenesis in *cyp26a1*–/–medaka, since the main RA degrading enzyme is absent. However, our data from the mutants demonstrate on the contrary that AM580 blocks germ cell differentiation, like in wildtype medaka. The lack of meiosis induction in the *cyp26a1* mutants could be explained by a possible gene compensation of *cyp26b1*. On the other side, our result is in line with the induction of *dmrt1a* and *amh* in wildtype embryos after RA treatments, indicating an activation of the male pathway, which is marked by a reduced germ cell proliferation and differentiation. On the other hand, our previous experiments demonstrated that increased cortisol levels induce masculinization of XX medaka by direct activation of the *dmrt1a* promoter (Adolfi et al., 2019). Hence, another explanation for the activation of *dmrt1a* after RA treatment could have been that this is simply due to a stress condition, since all embryos showed malformation after treatments with this morphogen. The treatments with both AM580 and Metyrapone (cortisol synthesis inhibitor) demonstrated that the germ cells respond to RA physiologically leading to increased meiosis and early oogenesis in females. Males, however, did not show oogenesis nor spermatogenesis stimulation after treatments at 1 dah. In mammals, DMRT1 allows Sertoli cells to participate in RA signaling while avoiding consequent cell fate reprogramming (Huang et al., 2017). At the time when meiosis initiates in females, in males *dmrt1bY* is expressed in the gonads (Nishimura et al., 2014), and possibly restrains the germ cells from entering differentiation. Hence, despite of mutating the main RA degrading enzyme, and treating the embryos with exogenous RA, the timing of meiosis entry is still different between males and females likely due to expression of *dmrt1bY, dmrt1a*, or even other male development promoting factors in the somatic gonads of males (Figure 5).

In summary, we showed that RA has an important role in regulating meiosis and gametogenesis but only after the sex determination stage. Exogenous treatments with ATRA and AM580 in wildtype embryos reduced the meiosis entry by activation of male-related genes, probably due to the stress conditions. Full knockouts of the main RA degrading Cyp26a1 led to an increase of meiosis in female embryos and to the regulation of genes related to gametogenesis and meiosis entry in adult gonads. In males, despite some mutants showing oocytes in the early gonad, the timing of meiosis entry is still later than in females. This makes us to suggest that in medaka the differential expression between male and female of sexual development related genes regulates the responsiveness of the germ cells to RA independent of *stra8* regulation. Hence, the sex determination network limits the action of RA to a time after the sex determination period.

## Data Availability Statement

The raw data supporting the conclusions of this article will be made available by the authors, without undue reservation.

## Ethics Statement

The animal study was reviewed and approved by Veterinary Office of the District Government of Lower Franconia, Germany (568/300-1870/13).

## Author Contributions

MA carried out the mutant line production, sampling, treatments, histology, imaging, molecular analysis, and drafted the manuscript. AH coordinated part of the study and helped to review the manuscript. AM-B extracted the RNA samples from gonads of wildtype medakas for sequencing. SK carried out the bioinformatic analyses of the transcriptomes. MR carried out part of the qRT-PCR and luciferase assay. DG designed and provided the *cyp26a1* TALEN plasmids for mRNA synthesis. MS defined and designed the study, coordinated all steps of the research, and reviewed all versions of the manuscript. All authors contributed to the article and approved the submitted version.

## Conflict of Interest

The authors declare that the research was conducted in the absence of any commercial or financial relationships that could be construed as a potential conflict of interest.

## Supplementary Material

Supplementary Figure 1 Responsiveness of HEK 293 cells to exogenous treatments with ATRA and AM580. Transfection of plasmids containing retinoic acid responsive elements (RARE) in HEK 293 cells resulted increased luciferase activity after treatments with both ATRA and AM580.

Supplementary Figure 2 Genomic structure of the medaka *cyp26a1* gene and mutant sequences induced by TALENs. **(A)** The *cyp26a1* TALENs were designed to target the second exon of the gene. The right binding site was located at the junction of exon 2 and intron 2. Underlined bases indicate the left and right recognition sequences of the TALENs. Forward and reverse primers were designed to amplify the fragment for sequencing. **(B)** Wildtype and mutant *cyp26a1s*equences. **(C)** Predicted protein sequences. Amino acid substitutions are labeled in red. Deletions are indicated by dashes. E, exon; F, forward; R, reverse; WT, wildtype.

Supplementary Figure 3 Morphology of adult gonads of wildtype and *cyp26a1*–/–medaka. Both mature wildtype **(A)** and mutant ovaries **(B)** presented big vitellogenic oocytes (Vo). Scale bar = 400 μm. In mature males, greater amounts of spermatozoa (star) are observed inside the testicular ducts in wildtype (**C**, scale bar = 50 μm) than in the mutant (**D**, scale bar = 100 μm).

Supplementary Table 1 Sequences of the oligos used in the present study.

Supplementary Table 2 List of genes strongly regulared in cyp26a1–/–(−2 < LogFC > 2).
