# Supplementary figures and images for: Crosstalk Between Retinoic Acid and Sex-Related Genes Controls Germ Cell Fate and Gametogenesis in Medaka
Source: Front Cell Dev Biol. 2021 Jan 18;8:613497. doi: 10.3389/fcell.2020.613497 (PMC7848095; doi:10.3389/fcell.2020.613497)

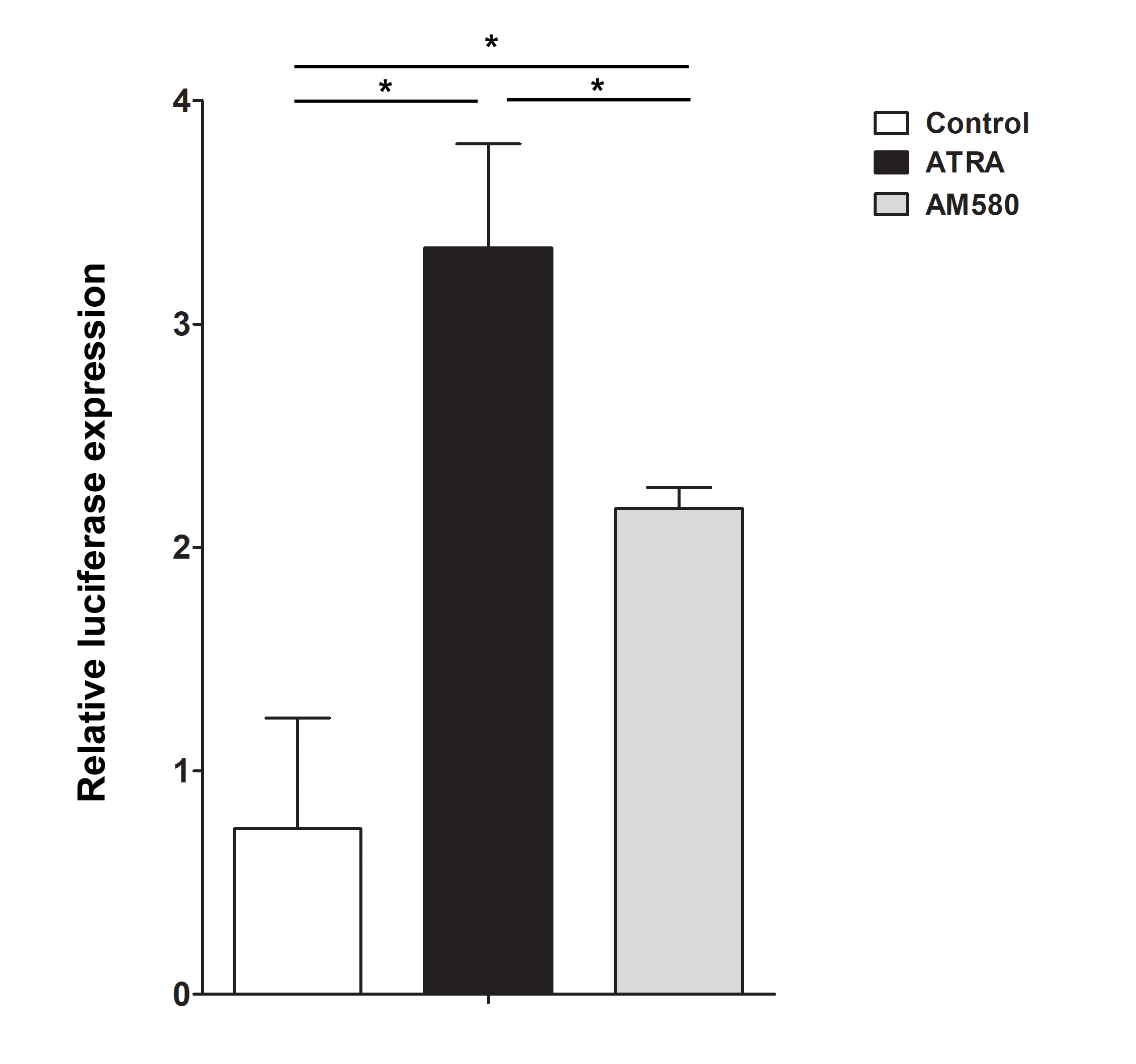

Supplement: Supplementary Figure 1 — Responsiveness of HEK 293 cells to exogenous treatments with ATRA and AM580. Transfection of plasmids containing retinoic acid responsive elements (RARE) in HEK 293 cells resulted increased luciferase activity after treatments with both ATRA and AM580. [file Image_1.TIF]

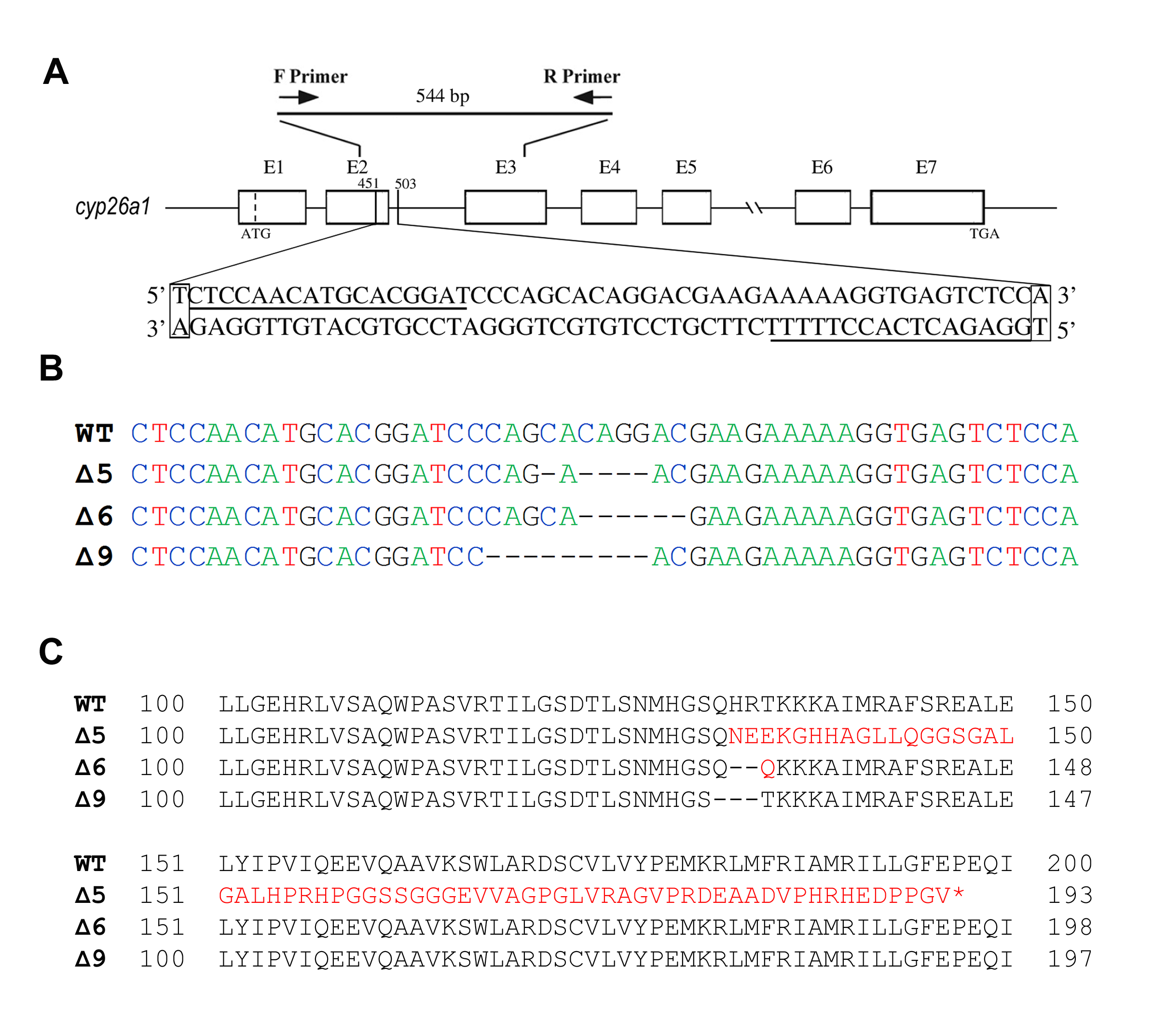

Supplement: Supplementary Figure 2 — Genomic structure of the medaka cyp26a1 gene and mutant sequences induced by TALENs. (A) The cyp26a1 TALENs were designed to target the second exon of the gene. The right binding site was located at the junction of exon 2 and intron 2. Underlined bases indicate the left and right recognition sequences of the TALENs. Forward and reverse primers were designed to amplify the fragment for sequencing. (B) Wildtype and mutant cyp26a1sequences. (C) Predicted protein sequences. Amino acid substitutions are labeled in red. Deletions are indicated by dashes. E, exon; F, forward; R, reverse; WT, wildtype. [file Image_2.TIF]

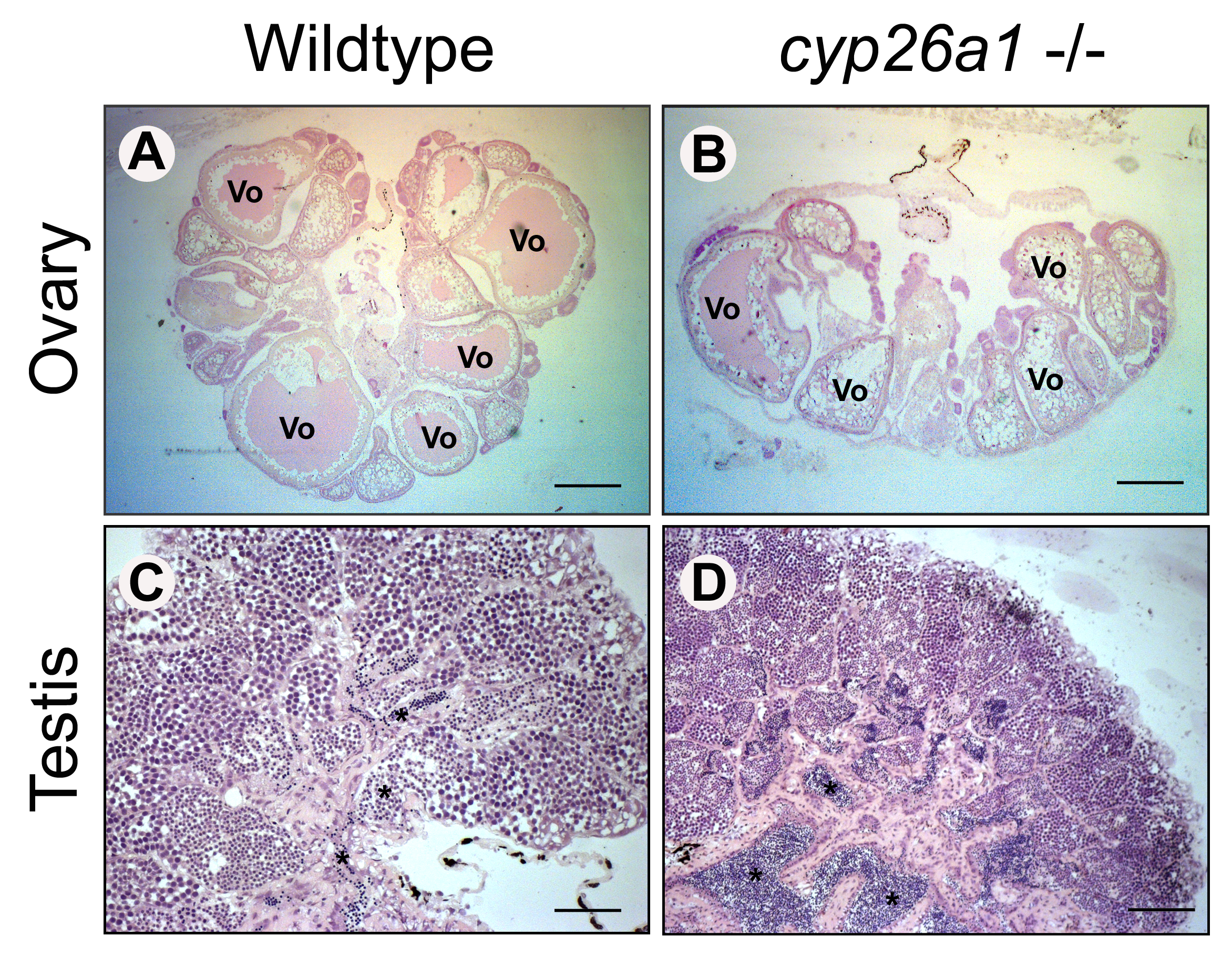

Supplement: Supplementary Figure 3 — Morphology of adult gonads of wildtype and cyp26a1–/–medaka. Both mature wildtype (A) and mutant ovaries (B) presented big vitellogenic oocytes (Vo). Scale bar = 400 μm. In mature males, greater amounts of spermatozoa (star) are observed inside the testicular ducts in wildtype (C, scale bar = 50 μm) than in the mutant (D, scale bar = 100 μm). [file Image_3.TIF]
